# Microbial enzymes: industrial progress in 21st century

**DOI:** 10.1007/s13205-016-0485-8

**Published:** 2016-08-19

**Authors:** Rajendra Singh, Manoj Kumar, Anshumali Mittal, Praveen Kumar Mehta

**Affiliations:** 1 Department of Biochemistry, VP Chest Institute, University of Delhi, Delhi, 110007 India; 2 Mill Hill Laboratory, Division of Structural Biology and Biophysics, The Francis Crick Institute, London, UK; 3 Department of Biotechnology and Food Engineering, Technion-Israel Institute of Technology, 32000 Haifa, Israel

**Keywords:** Microorganisms, Enzymes, Bioconversion, Application, Industry

## Abstract

**Abstract:**

Biocatalytic potential of microorganisms have been employed for centuries to produce bread, wine, vinegar and other common products without understanding the biochemical basis of their ingredients. Microbial enzymes have gained interest for their widespread uses in industries and medicine owing to their stability, catalytic activity, and ease of production and optimization than plant and animal enzymes. The use of enzymes in various industries (e.g., food, agriculture, chemicals, and pharmaceuticals) is increasing rapidly due to reduced processing time, low energy input, cost effectiveness, nontoxic and eco-friendly characteristics. Microbial enzymes are capable of degrading toxic chemical compounds of industrial and domestic wastes (phenolic compounds, nitriles, amines etc.) either via degradation or conversion. Here in this review, we highlight and discuss current technical and scientific involvement of microorganisms in enzyme production and their present status in worldwide enzyme market.

**Graphical abstract:**

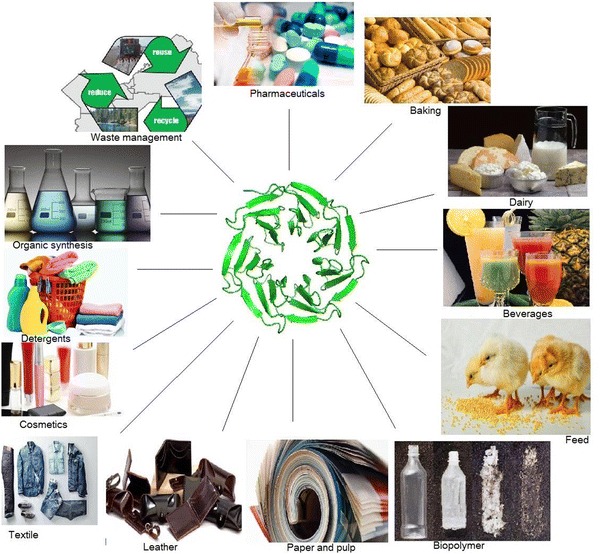

## Introduction

Microbes have been utilized since ancient human civilization with first reported commercial application of yeast to produce alcoholic beverages from barley by the Babylonians and Sumerians as early as 6000 BC. The microbial enzymes have gained recognition globally for their widespread uses in various sectors of industries, e.g., food, agriculture, chemicals, medicine, and energy. Enzyme mediated processes are rapidly gaining interest because of reduced process time, intake of low energy input, cost effective, nontoxic and eco-friendly characteristics (Li et al. 2012; Choi et al. 2015). Moreover, with the advent of recombinant DNA technology and protein engineering a microbe can be manipulated and cultured in large quantities to meet increased demand (Liu et al. 2013). Associated driving factors that motivate the use of microbial enzymes in industrial applications are increasing demand of consumer goods, need of cost reduction, natural resources depletion, and environmental safety (Choi et al. 2015). Global market for industrial enzymes was estimated about $4.2 billion in 2014 and expected to develop at a compound annual growth rate (CAGR) of approximately 7 % over the period from 2015 to 2020 to reach nearly $6.2 billion ( 2015b: Industrial Enzyme Market). Enzymes are biological molecules, proteinaceous in nature with the exception of catalytic RNA molecules (ribozymes), and act as catalyst to support almost all of the chemical reactions required to sustain life (Cech and Bass 1986). Enzymes are highly specific; only accelerate the rate of particular reaction by lowering the activation energy without undergoing any permanent change in them, and therefore, are vital biomolecules that support life (Fersht 1985; Piccolino 2000; Aldridge 2013). They require typically milder condition of temperature and pressure for catalyzing reactions, and are used as an alternative to hazardous chemical pollutant owing to their biodegradable and nontoxic nature (Mojsov 2011; Illanes et al. 2012; Choi et al. 2015). In addition to advantages of enzymes over conventional methods, there are some drawbacks of using enzymes in healthcare and other industries. For many mammalian enzymes, 37°C and 7.4 are the optimal temperature and pH, respectively, and their activity is highly sensitive to any change in these parameters. Higher temperature (>40°C), and a large deviation from the physiological pH (7.4) lead to their denaturation, which limits the use of these macromolecules in non-physiological conditions. Additionally, they are susceptible to substrate or product inhibition and their products may cause allergic reactions. The high cost of isolation and purification of enzymes and their difficult recovery for subsequent reuse may discourage their use (Johannes et al. 2006).

Enzymes are large macromolecules composed of polymers of amino acids connected by amide bonds, ranging from kilodalton (insulin) to megadalton (ribosome) in molecular mass. Catalytic site of these macromolecules is often buried deep within hydrophobic pockets, which determines the specificity for their substrate. This specificity of enzyme to catalyze reactions between one types of chemical compound over the other provides the basis of its classification and name. With the great advancement achieved in the area of biochemistry after 1940, a large number of enzymes were isolated and characterized, and therefore, it was necessary to regulate the enzyme nomenclature. Thus, International Union of Biochemistry and Molecular Biology (IUBMB) in consultation with International Union for Pure and Applied Chemistry (IUPAC) established an Enzyme Commission (EC) to be in charge of guiding the naming and systematic classification for enzymes (Liese et al. 2006). According to the type of reaction catalyzed, the enzyme commission has classified the enzymes into six main classes, mentioned in Table 1.

**Table 1:** Enzyme classes, types of reactions and corresponding enzymes

S. no.	Class	Reactions	Enzymes
1	Oxidoreductases	Transfer of hydrogen or oxygen or electrons between molecules	Dehydrogenases, oxidases, oxygenases, peroxidases
2	Transferases	Transfer of groups of atoms from one molecule to another	Fructosyltransferases, transketolases, acyltransferases, transaminases
3	Hydrolases	Hydrolytic cleavage of bonds	Proteases, amylases, acylases, lipases, phosphatases, cutinases
4	Lyases	Non-hydrolytic cleavage by elimination or addition reactions	Pectate lyases, hydratases, dehydratases, decarboxylases, fumarase, argino succinase
5	Isomerases	Transfer of group from one position to another within one molecule	Isomerases, epimerases, racemases
6	Ligases	Covalent joining of two molecules coupled with the hydrolysis of an energy rich bond in ATP or similar triphosphates	Synthetases, ligases

Microorganisms are favored sources for industrial enzymes due to easy availability, and fast growth rate. Genetic changes using recombinant DNA technology can easily be done on microbial cells for elevated enzyme production and scientific development (Illanes et al. 2012). Production of microbial enzymes is a necessary event in the industrial sectors, due to the high and superior performances of enzymes from different microbes, which work well under a wide range of varied physical and chemical conditions. Further, microbial enzymes are used in the treatment of health disorders associated with deficiency of human enzymes caused by genetic problems (Vellard 2003; Anbu et al. 2015). For instance, patients with inherited congenital sucrase-isomaltase deficiency are unable to digest sucrose, and therefore, sacrosidase (β-fructofuranoside fructohydrolase, EC 3.2.1.26) enzyme is given orally to facilitate digestion of sucrose (Treem et al. 1999). In addition, phenylalanine ammonia lyase (EC 4.3.1.24) is used to degrade phenylalanine in genetic phenylketonuria disorder (Sarkissian et al. 1999).

**Fig. 1:**
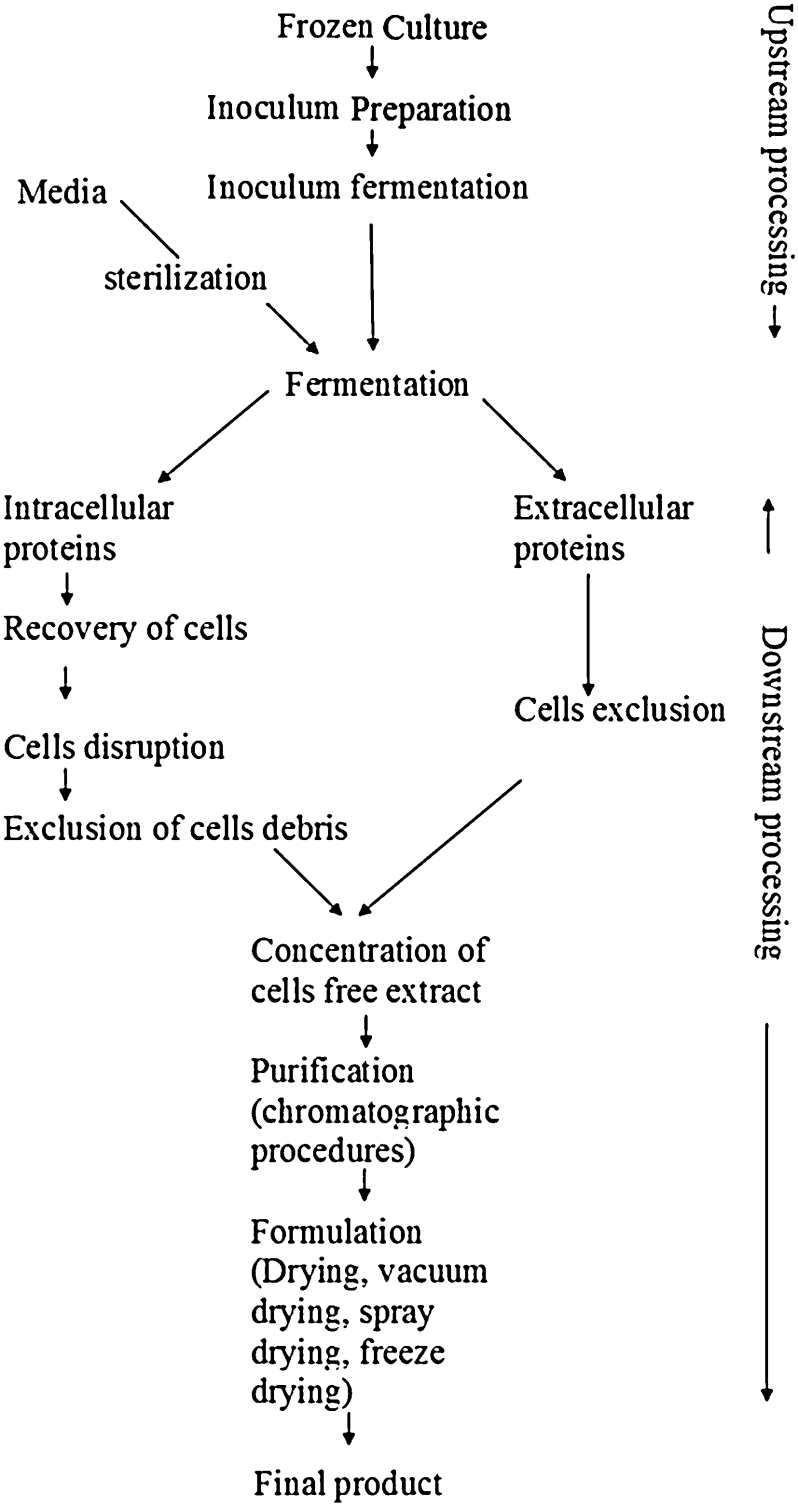
Industrial production of enzymes

The extensive application of microbes in different bioprocess is used to deliver a variety of products in applied industries. Table 2 summarizes several applications of microorganisms to deliver a variety of products. The Schematic representation of industrial production of microbial enzymes has been shown in Fig. 1.

**Table 2:** Industrial applications of microbial enzymes

Industry	Enzyme	Function	Microorganisms
Dairy	Acid proteinase	Milk coagulation	*Aspergillus* sp.
	Neutral proteinase	Faster cheese ripening, debittering	*Bacillus subtilis, A. oryzae*
	Lipase	Faster cheese ripening, flavor customized cheese,	*Aspergillus niger, A. oryzae*
	Lactase (β-galactosidase)	Lactose reduced milk and whey products	*Escherichia coli, Kluyveromyces* sp.
	Aminopeptidase	Faster cheese ripening	*Lactobacillus* sp.
	catalase	Cheese processing	*Aspergillus niger*
	Transglutaminase	Protein cross linking	*Streptomyces* sp.
Baking	Amylase	Flour adjustment, bread softness	*Aspergillus* sp*., Bacillus* sp.
	Maltogenic α-Amylase	Enhance shelf life of breads	*Bacillus stearothermophilus*
	Xylanase	Dough conditioning	*Aspergillus niger*
	Lipase	Dough stability and conditioning	*Aspergillus niger*
	Glucose oxidase	Dough strengthening	*Aspergillus niger, Penicillium chrysogenum*
	Transglutaminase	Laminated dough strength	*Streptoverticillium* sp., *streptomyces* sp.
Beverage	Pectinase	Depectinization	*Aspergillus oryzae, Penicillium funiculosum*
	Glucose oxidase	Oxygen removal from beer	*Aspergillus niger*
	Cellulase	Fruit liquefaction	*Aspergillus niger, Trichoderma atroviride*
	α-Amylase	Starch hydrolysis	*Bacillus, Aspergillus*
	β-Amylase	Starch hydrolysis	*Bacillus, Streptomyces, Rhizopus*
	β-Glucanase	Restrict haze formation	*Bacillus subtilis, Aspergillus* spp.
	protease	Restrict haze formation	*Aspergillus niger*
	Pullulanase	Starch saccharification	*Bacillus* sp*., Klebsiella* sp.
	Naringinase	Debittering	*Aspergillus niger*
	limoninase	Debittering	*Aspergillus niger, A. oryzae*
	Aminopeptidases	Protein breakdown during mashing	*Lactobacillus brevis, L. plantarum*
Animal feed	Phytase	Hydrolyze phytic acid to release phosphorous	*Aspergillus niger*
	Xylanase	Enhanced digestibility of starch	*Aspergillus* sp*., Bacillus* sp.
	β-glucanase	Digestive aid	*Aspergillus niger*
Pulp and paper	Lipase	Pitch control	*Candida Antarctica*
	Protease	Biofilm removal	*Bacillus subtilis*
	Amylase	Deinking, drainage improvement	*Bacillus licheniformis*
	Xylanase	Bleach boosting	*Trichoderma reesei, Thermomyces lanuginosus, Aureobasidium pullulans*
	Laccase	Non-chlorine bleaching, delignification	*Bacillus subtilis*
	Cellulase	Deinking, drainage improvement	*Bacillus* sp*., Aspergillus niger*
Polymer	Lipase	Polycondensation, ring-opening polymerization of lactones, carbonates	*Candida Antarctica*
	Laccase	Polymerization of bisphenol A	*Trametes hirsuta*
	Glucose oxidase	Polymerization of anilines	*Aspergillus niger, Penicillium chrysogenum*
	Transglutaminase	Crosslinking of protein	*Streptomyces mobaraensis*
	Tyrosinase	Polymerization of lignin and chitosan	*Trichoderma reesei*
Detergent	Amylase	Carbohydrate stain removal	*Aspergillus* sp*., Bacillus subtilis*
	Lipase	Fat stain elimination	*Aspergillus oryzae, A. flavus,*
	Protease	Protein stain removal	*Aspergillus oryzae, Bacillus subtilis*
	Cellulase	Color clarification	*Aspergillus niger, Bacillus* sp.
	Cutinase	Triglyceride removal	*Fusarium solani f. pisi*
	Mannanase	Mannan spot removal	*Bacillus* sp.
Leather	Alkaline protease	Dehairing, bating	*Alcaligenes faecalis*
	Neutral Protease	Dehairing, soaking	*Aspergillus niger, A. flavus, Bacillus subtilis*
	Lipase	Degreasing	*Aspergillus oryzae, A. flavus,*
	Amylase	Fiber splitting	*Aspergillus* sp*., Bacillus subtilis*
Cosmetics	Superoxide dismutase	Free radical scavenging, skin care	*Corynebacterium* *Glutamicum, Lactobacillus plantarum*
	Protease	Removal of dead skin	*Aspergillus niger, A. flavus, Bacillus subtilis*
	Endoglycosidase	Teeth and gum tissue care	*Mucor hiemalis*
	laccase	Hair dye	*Bacillus subtilis, Trametes versicolor*
	lipase	Skin care	*Aspergillus oryzae, A. flavus*
Organic synthesis	Lipase	Synthesis of pharmaceuticals, polymers, biodiesels, biosurfactants	*Aspergillus oryzae, A. flavus*
	Glycosyl tranferase	Synthesis of oligosaccharides	*Bacillus* sp.
	Nitrile hydratase	Synthesis of acrylamide, butyramide, nicotinamide	*Rhodococcus rhodochrous* PA-34*, Bacillus* sp*. APB*-*6*
	Glucose isomerase	Production of High fructose corn syrup	*Corynebacterium* sp*., streptomyces murinus*
	Acyltransferase	Synthesis of hydroxamic acids	*Bacillus* sp. APB-6
	Laccase	Production of textile dyes, cosmetic pigments, flavor agents, and pesticides	*Trametes versicolor, Bacillus subtilis*
Waste management	Amidase	Degradation of nitriles containing wastes	*Rhodococcus erythropolis*
	Amylase	Bioremediation of vegetables wastes	*B. licheniformis, Aspergillus* sp.
	Amyloglucosidase	Starch hydrolysis for bioremediation	*Aspergillus niger*
	Lipase	Degradation of crude oil hydrocarbons	*Aspergillus oryzae, Candida tropicalis*
	Nitrile hydratase	Degradation of nitriles containing wastes	*Rhodococcus* sp.
	Protease	Bioremediation of keratinic wastes	*Chrysosporium keratinophilum*
	Laccase	Degradation of waste containing olefin unit, polyurethane and phenolic compounds	*Trametes versicolor*
	Cutinase	Degradation of plastics, Polycaprolactone	*Fusarium solani f. pisi*
	Manganese peroxidase	Degradation of phenolic compounds	*Phanerochaete chrysosporium, Coprinus cinereus*
	Lignin peroxidase	Degradation of phenolic compounds	*Phanerochaete chrysosporium, Coprinus cinereus*
	Oxygenase	Degradation of halogenated contaminants	*Pseudomonas* sp*., Rhodococcus* sp.

Enzymes, particularly of microbial origin, can be cultured largely by gene manipulations, as per the need for industrial applications. Applications of microbial enzymes in food, pharmaceutical, textile, paper, leather, and other industries are numerous and increasing rapidly over conventional methods due to less harm to the environment, greater efficiency, and the higher quality products (Jordon 1929; Kamini et al. 1999; Gurung et al. 2013).

In this review, the attentions are given to present a succinct role of all microbial enzymes involved in various fields of technical applications, such as feed industry, food processing and cosmetics. Furthermore, efforts are made to draw a simple and clear scenario about the industrial structure of global enzyme market.

## The use of enzymes in industrial processes

Enzymes are used in industrial processes, such as baking, brewing, detergents, fermented products, pharmaceuticals, textiles, leather processing. Here are a range of processes showing how enzymes are used.

### Pharmaceutical and analytical industry

Enzymes have many significant and vital roles in the pharmaceutical and diagnostic industries. These are extensively used as therapeutic drugs in health issues associated with enzymatic deficiency and digestive disorders, and in diagnostic procedures such as ELISA and diabetes testing kits (Mane and Tale 2015).

Enzyme applications in medicine are as extensive as in industry and are growing rapidly. At present, most prominent medical uses of microbial enzymes are removal of dead skin, and burns by proteolytic enzymes, and clot busting by fibrinolytic enzymes. Nattokinase (EC 3.4.21.62), a potent fibrinolytic enzyme, is a promising agent for thrombosis therapy (Sumi et al. 1987; Cho et al. 2010). Acid protease, dextranase (EC 2.4.1.2) and rhodanase (EC 2.8.1.1) may be used to treat alimentary dyspepsia, tooth decay and cyanide poisoning, respectively (Okafor 2007). Lipases (EC 3.1.1.3) are the most frequently used enzymes in the organic synthesis and are used in the synthesis of optically active alcohols, acids, esters, and lactones (Cambou and Klibanov 1984; Saxena et al. 1999). Microbial lipases and polyphenol oxidases (EC 1.10.3.2) are involved in the synthesis of (2R,3S)-3-(4-methoxyphenyl) methyl glycidate   (an intermediate for diltiazem) and 3, 4-dihydroxylphenyl alanine (DOPA, for treatment of Parkinson’s disease), respectively (Faber 1997). Tyrosinase (EC 1.14.18.1), an important oxidase enzyme, is involved in melanogenesis and in the production of l-Dihydroxy phenylalanine (L-DOPA). L-DOPA is used as a precursor for the production of dopamine which is a potent drug for the treatment of Parkinson’s disease and to control the myocardium neurogenic injury (Ikram-ul-Haq et al. 2002; Zaidi et al. 2014). Chitosanase (EC 3.2.1.132) catalyze hydrolysis of chitosan to biologically active chitosan oligosaccharides (COS), which is used as antimicrobial, antioxidant, lowering of blood cholesterol and high blood pressure, controlling arthritis, protective effects against infections and improving antitumor properties (Kim and Rajapakse 2005; Ming et al. 2006; Zhang et al. 2012; Thadathil and Velappan 2014). Applications of microbial enzymes for different health problems are illustrated in Table 3 (Devlin 1986; Kaur and Sekhon 2012; Sabu 2003; Vellard 2003; Mane and Tale 2015).

**Table 3:** Some therapeutic applications of microbial enzymes

Treatment	Enzymes	Microorganisms
Antitumor	l-Asparaginase, l-glutaminase, l-tyrosinase, galactosidase	*Escherichia coli, Pseudomonas acidovorans, Beauveria bassiana, Acinetobacter*
Antiinflammatory	Superoxide dismutase, Serrapeptase	*Lactobacillus plantarum, Nocardia* sp*., Mycobacterium* sp*., Corynebacterium Glutamicum,*
Anticoagulants	Streptokinase, urokinase	*Streptococci* sp*., Bacillus subtilis*
Antibiotic synthesis	Penicillin oxidase, rifamycin B oxidase	*Penicillium* sp.
Antioxidants	Superoxide dismutases, glutathione peroxidases, catalase	*Lactobacillus plantarum, Corynebacterium glutamicum*
Skin ulcers	Collagenase	*Clostridium perfringens*
Detoxification	Laccase, rhodanese	*Pseudomonas aeruginosa*
Antibiotic resistance	β-Lactamase	*Klebsiella pneumonia, Citrobacter freundii, Serratia marcescens*
Antiviral	Ribonuclease, Serrapeptase	*Saccharomyces cerevisiae*
Gout	Uricase	*Aspergillus flavus*
Digestive disorders	α-Amylase, lipase	*Bacillus* spp*., Candida lipolytica, A. oryzae*
Cyanide poisoning	Rhodanase	*Sulfobacillus sibiricus*

The extensive utilization of enzymes for scientific and analytical purposes is used to estimate the concentration of substrates and to determine the catalytic activity of enzymes present in biological samples. Advances in the enzyme technology have replaced or minimized the use to harmful radioactive elements in different immunoassays, which are used for the determination of a variety of proteins and hormones (Palmer 2001).

Furthermore, enzymes are used in clinical diagnostic for the quantitative determination of diabetes and other health disorders, for example, glucose oxidase (EC 1.1.3.4) for glucose; urease (EC 3.5.1.5) and glutamate dehydrogenase (EC 1.4.1.2) for urea; lipase, carboxyl esterase, and glycerol kinase for triglycerides; urate oxidase (EC 1.7.3.3) for uric acid; creatinase (EC 3.5.3.3) and sarcosine oxidases (EC 1.5.3.1) for creatinine (Dordick 2013; Le Roes-Hill and Prins 2016). Cholesterol oxidase (EC 1.1.3.6) has also been reported for useful biotechnological applications in the detection and conversion of cholesterol. Putrescine oxidase (EC 1.4.3.10) is used to detect biogenic amines, such as putrescine, a marker for food spoilage (Le Roes-Hill and Prins 2016).

Enzymes are indispensable in nucleic acid manipulation for research and development in the field of genetic engineering, such as restriction endonucleases are used for site specific cleavage of DNA for molecular cloning (Newman et al. 1995) and DNA polymerases for the DNA amplification by polymerase chain reaction (PCR).

### Food industry

United Nation Department of Economic and Social Affairs (UNDESA) estimates that world population is predicted to grow from 6.9 billion to 9.1 billion and food demand is expected to increase by 70 % by 2050 (http://www.un.org/waterforlifedecade/food_security.shtml). Quality food supply issue can be addressed by the application of enzymes in the food industry. These biomolecules are efficiently involved in improving food production and components, such as flavor, aroma, color, texture, appearance and nutritive value (Neidleman 1984). The profound understanding of the role of enzymes in the food manufacturing and ingredients industry have improved the basic processes to provide better markets with safer and higher quality products. Furthermore, the enzymes gained interest in new areas such as fat modification and sweetener technology (Li et al. 2012). Currently, the food and beverage segment dominated the industrial enzyme market and it is projected to reach a value of $2.3 billion by 2020 (2015c: Industrial Enzymes Market). In beverage and food industry, enzymes are added to control the brewing process and produce consistent, high-quality beer; to enhance the functional and nutritional properties of animal and vegetables proteins by the enzymatic hydrolysis of proteins, for higher juice yield with improved color and aroma.

The application of enzymes in food industry is segmented into different sectors, such as baking, dairy, juice production and brewing. Worldwide, microbial enzymes are efficiently utilized in bakery—the principal application market in food industry—to improve dough stability, crumb softness and structure, and shelf life of products. Increased uses of microbial enzymes in cheese processing are largely responsible for the use of enzymes in dairy industry, which is the next largest application industry followed by the beverages industry.

#### Baking industry

Baking enzymes are used for providing flour enhancement, dough stability, improving texture, volume and color, prolonging crumb softness, uniform crumb structure and prolonging freshness of bread. To meet rising demand for quality, enzymes are seen as natural solutions in today’s baking market. The baking enzymes industry is expected to reach $695.1 million by 2019 growing at a CAGR 8.2 % from 2013 to 2019 (2014a: Baking Enzymes Market).

Bread making is one of the most common food processing techniques globally. The use of enzymes in bread manufacturing shows their value in quality control and efficiency of production. Amylase, alone or in combination with other enzymes, is added to the bread flour for retaining the moisture more efficiently to increase softness, freshness and shelf life. Additionally, lipase and xylanase (EC 3.2.1.8) are used for dough stability and conditioning while glucose oxidase and lipoxygenase added to improve dough strengthening and whiteness. Transglutaminase (EC 2.3.2.13) is used in baking industry to enhance the quality of flour, the amount and texture of bread and the texture of cooked pasta (Kuraishi et al. 1997; Moore et al. 2006; Kieliszek and Misiewicz 2014). Lipases are also used to improve the flavor content of bakery products by liberating short-chain fatty acids through esterification and to prolong the shelf life of the bakery products (Andreu et al. 1999; Dauter et al. 1999; Monfort et al. 1999; Collar et al. 2000; Kirk et al. 2002; Fernandes 2010; Li et al. 2012; Adrio and Demain 2014).

#### Dairy industry

Dairy enzymes, an important segment of food enzyme industry, are used for the development and enhancing organoleptic characteristics (aroma, flavor and color) and higher yield of milk products. The use of enzymes (proteases, lipases, esterases, lactase, aminopeptidase, lysozyme, lactoperoxidase, transglutaminase, catalase, etc.) in dairy market is well recognized and varies from coagulant to bio-protective enzyme to enhance the shelf life and safety of dairy products. Dairy enzymes are used for the production of cheese, yogurt and other milk products (Pai 2003; Qureshi et al. 2015).

Rennet, a combination of chymosin and pepsin, is used for coagulation of milk into solid curds for cheese production and liquid whey. Currently, approximately 33 % of global demand of cheese produced using microbial rennet. Other proteases find applications for accelerated cheese processing and in reduction of allergenic properties of milk products (Qureshi et al. 2015). Currently, lipases are involved in flavor improvement, faster cheese preparation, production of customized milk products, and lipolysis of milk fat (Sharma et al. 2001; Ghosh et al. 1996). Transglutaminase catalyzes polymerization of milk proteins and improves the functional properties of dairy products (Rossa et al. 2011; Kieliszek and Misiewicz 2014).

Lactose intolerance is the lack of ability of human being to digest lactose due to deficiency of lactase enzyme. Lactase (β-galactosidase, EC 3.2.1.23) catalyzes hydrolysis of lactose to glucose and galactose, and therefore, is used as a digestive aid and to enhance the solubility and sweetness in milk products (Soares et al. 2012; Qureshi et al. 2015). It is required to minimize or removal of lactose content of milk products for lactose-intolerant people to prevent severe tissue dehydration, diarrhea, and sometimes fatal consequences (Kardel et al. 1995; Pivarnik et al. 1995; Mahoney 1997).

#### Beverages industry

The beverage industry is divided into two major groups and eight sub-groups. The nonalcoholic group contains soft drink and syrup, packaged water, fruit juices along with tea and coffee industry. Alcoholic group comprised distilled spirits, wine and beer (Encyclopedia of Occupational health and safety). Industrial enzymes are used in breweries as processing aids and to produce consistent and high-quality products. In the brewing industries, microbial enzymes are used to digest cell wall during extraction of plant material to provide improved yield, color, and aroma and clearer products (Kårlund et al. 2014).

The enzyme applications are an integrating ingredient of the current fruit and vegetable juice industry. Enzymes are used in fruit and vegetable juice industry as processing aids to increase the efficiency of operation, for instance, peeling, juicing, clarification, extraction and improve the product quality (Law 2002). Application of cellulases, amylases, and pectinases during fruit juice processing for maceration, liquefaction, and clarification, improve yield and cost effectiveness (Kumar 2015; Garg et al. 2016). The quality and stability of juices manufactured are enhanced by the addition of enzymes. Enzymes digest pectin, starch, proteins and cellulose of fruits and vegetables and facilitate improved yields, shortening of processing time and enhancing sensory characteristics (Mojsov 2012). Amylases are used for clarification of juices to maximize the production of clear or cloudy juice (Vaillant et al. 2001; Sivaramakrishnan et al. 2006). Cellulases and pectinases are used to improve extraction, yield, cloud stability and texture in juices (Bhat 2000; Kashyap et al. 2001; Garg et al. 2016). Naringinase (EC 3.2.1.40) and limoninase, debittering enzymes, hydrolyze bitter components and improves the quality attributes of citrus juices (Hotchkis and Soares 2000; Li et al. 2012). Pectin, a structural heteropolysaccharide, present in nearly all fruits is required to be maintained to regulate cloudiness of juices by polygalacturonase (EC 3.2.1.15), pectin esterases (EC 3.1.1.11), pectin lyase (EC 4.2.2.10) and various arabanases (Kashyap et al. 2001; Yadav et al. 2001).

Microbial amylases may be utilized in the distilled alcoholic beverages to hydrolyze starch to sugars prior to fermentation and to minimize or remove turbidities due to starch. The application of enzymes to hydrolyze unmalted barley and other starchy adjuncts facilitate in cost reduction of beer brewing. In brewing, development of chill-hazes in beer may be control by the addition of proteases (Okafor 2007).

### Feed industry

To meet the continuously increasing worldwide demand of milk and meat consumption, growth of feed enzymes occurred steadily. The use of enzymes in animal diets initiated in the 1980s and exploded in the 1990s. Feed enzymes are gaining importance as they can increase the digestibility of nutrients and higher feed utilization by animals (Choct 2006). The global market for feed enzymes was estimated $899.19 million in 2014 and expected to reach nearly $1.3 billion by 2020, at a CAGR of 7.3 % from 2015 to 2020 (2015a: Feed Enzyme Market).

Feed enzymes may be used in animal diet formulation. For instance, these are added to degrade specific feed components which are otherwise harmful or no nutritional value. In addition, the protein dietary value of feeds available for poultry may also be enhanced by the application of feed enzymes (Collection of information on enzymes 2002). Feed enzymes mainly used for poultry are phytases, proteases, α-galactosidases, glucanases, xylanases, α-amylases, and polygalacturonases (Walsh et al. 1993; Chesson 1993; Bhat 2000; Adrio and Demain 2014). The phytase, largest enzyme segment in the feed industry, is used to utilize natural phosphorous bound in phytic acid in cereal-based feed (Lei and Stahl 2000; Bhat 2000; Frias et al. 2003). Monogastric animals are unable to digest plant based feeds containing high amount of cellulose and hemicelluloses. Xylanase and β-glucanase are added to their feeds as these enzymes fully degrade and digest high amount of starch (Bhat 2000; Kirk et al. 2002). Proteases are also used in animal feeds to overcome anti-nutritional factors by degrading proteins into their constituent amino acids. Apart from improving the nutritional value of feed for better feed conversion by the animals, these feed enzymes are gaining importance for their role in feed cost reduction and meat quality improvement (Lei and Stahl 2001; Adrio and Demain 2014).

### Polymer industry

To meet the increased consumption of polymers and the growing concern for human health and environmental safety has led to the utilization of microbial enzymes for synthesis of biodegradable polymer. In vitro enzyme catalyzed synthesis of polymer is an environmental safe process having several advantages over conventional chemical methods (Vroman and Tighzert 2009; Kadokawa and Kobayashi 2010). Biopolymers are environmentally friendly materials as these are synthesized from renewable carbon sources via biological processes, degrade biologically after use and return to the natural environment as renewable resources, such as CO_2_ and biomass (Hiraishi and Taguchi 2009). Biopolymers, such as polyesters, polycarbonates and polyphosphates are used in various biomedical applications, e.g., orthopedic devices, tissue engineering, adhesion barriers, control drug delivery, etc. (Gunatillake and Adhikari 2003; Ulery et al. 2011).

The biopolymers market is growing at a CAGR of 14.5 % due to high penetration of materials in industries like medical, packaging, appliances, automotive, electronics, and furniture and the market is expected to reach nearly $3.6 billion by 2018 ( 2014b: Bioplastics & Biopolymers Market). Increasing demand of packaging materials and environmental safety can be addressed by the biodegradable polymers. Lipases catalyze the polymerization of lactones, cyclic diesters and cyclic carbonates to produce polyesters or polycarbonates (Kobayashi 2010). Lipase catalyzed polymerization is an eco-friendly technique for the preparation of useful polyesters by polycondensation as well as poly-addition reactions (Vroman and Tighzert 2009). The other enzymes involved in biopolymer industries are laccase (EC 1.10.3.2), peroxidase and transglutaminase for forming cross-links in biopolymers to produce materials in situ by means of polymerization processes (Gurung et al. 2013).

### Paper and Pulp industry

With increasing awareness of sustainability issues, uses of microbial enzymes in paper and pulp industry have grown steadily to reduce adverse effect on ecosystem. The utilization of enzymes reduce processing time, energy consumption and amount of chemicals in processing. Enzymes are also used to enhance deinking, and bleach in paper and pulp industry and waste treatment by increasing biological oxygen demand (BOD) and chemical oxygen demand (COD) (Srivastava and Singh 2015). Xylanases and ligninases are used in paper and pulp industries to augment the value of the pulp by removing lignin and hemicelluloses (Maijala et al. 2008). In these industries, amylases uses include starch coating, deinking, improving paper cleanliness and drainage improvement (Kuhad et al. 2011). Lipases are employed for deinking and enhancing pitch control while cellulases are used for deinking, improving softness and drainage improvement (Kirk and Jeffries^1996^). Cellulase has also been used for the development of the bioprocess for recycling of used printed papers (Patrick 2004). The application of laccase is an alternative to usage and requirement of large amount of chlorine in chemical pulping process; subsequently, reduce the waste quantity that causes ozone depletion and acidification (Fu et al. 2005). Moreover, mannases are used for degrading glucomannan to improve brightness in paper industry (Clarke et al. 2000).

### Leather industry

The leather industry is more customary, and therefore, discharges and waste disposed from different stages of leather processing are causing severe health hazards and environmental problems (Choudhary et al. 2004). The biodegradable enzymes are efficient alternative to improve the quality of leather and help to shrink waste (Adrio and Demain 2014). The initial attempt for application of enzyme in leather industry was made for dehairing process, the largest process in leather preparation which require bulk amount of enzymes like proteases, lipases and amylases (Sankaran 1995; Bailey 1992; Raju et al. 1996). Enzymatic dehairing applications are attractive because it can preserve the hair and contribute to fall in the organic load released into the effluent. Enzymatic dehairing processes minimize or eliminate the dependence on harmful chemicals, such as sulfide, lime and amines (Green 1952; Money 1996; de-Souza and Gutterres 2012).

Enzymes are required for facilitating procedure and enhancing leather quality during different stages in leather processing, such as, curing, soaking, liming, dehairing, bating, picking, degreasing and tanning (Mojsov 2011). The enzymes used in leather industries are alkaline proteases, neutral proteases, and lipases. Alkaline proteases are used to remove non fibrillar proteins during soaking, in bating to make leather soft, supple and pliable. Neutral and alkaline proteases, both are used in dehairing to reduce water wastage (Rao et al. 1998). In addition to this, lipases are used during degreasing to remove fats (Choudhary et al. 2004; Sharma et al. 2001). The advantages of using enzymes instead of chemicals in liming are stainless pelt, reduced odor, low BOD and COD in effluents, and improved hair recovery (Bhatia 2003).

### Textile industry

The textile industry is responsible for vast generation of waste from desizing of fabrics, bleaching chemicals and dye is one of the largest contributors to environmental pollution (Ahuja et al. 2004). In such industries, enzymes are used to allow the development of environmentally friendly technologies in fiber processing and strategies to improve the final product quality (Choi et al. 2015). The main classes of enzymes involved in cotton pre-treatment and finishing processes are hydrolase and oxidoreductase. The group of hydrolase includes amylase, cellulase, cutinase, protease, pectinase and lipase/esterase, which are involved in the biopolishing and bioscouring of fabric, anti-felting of wool, cotton softening, denim finishing, desizing, wool finishing, modification of synthetic fibers, etc. (Araujo et al. 2008; Chen et al. 2013). Oxidoreductase, other group of enzyme, includes catalase, laccase, peroxidase, and ligninase, which are involved in bio-bleaching, bleach termination, dye decolorization, fabric, wool finishing, etc. (Mojsov 2011). A brief detail of applications of enzymes in textiles industries are shown in Table 4.

**Table 4:** Uses of enzymes in textile industry

Enzyme	Use	Microorganisms
Amylase	Desizing	*Bacillus* sp*., B. licheniformis*
Cellulose	Cotton softening, denim finishing	*Aspergillus niger, Penicillium funiculosum*
Catalase	Bleach termination	*Aspergillus* sp.
Laccase	Non-chlorine Bleaching, fabric dyeing	*Bacillus subtilis*
Pectate lyase	Bioscouring	*Bacillus* sp.*, Pseudomonas* sp.
Amylase	Desizing	*Bacillus* sp*., B. licheniformis*
Cellulose	Cotton softening, denim finishing	*Aspergillus niger, Penicillium funiculosum*
Protease	Removal of wool fiber scales, degumming of silk	*Aspergillus niger, B. subtilis*
Lipase	Removal of size lubricants, denim finishing,	*Candida Antarctica*
Ligninase	Wool finishing	*Trametes versicolor, Phlebia radiata*
Collagenase	Wool finishing	*Clostridium histolyticum*
Cutinase	Cotton scouring, synthetic fiber modification	*Pseudomonas mendocina, Fusarium solani pisi, Thermomonospora fusca*

### Enzymes in cosmetics

The applications of enzymes in cosmetics have been continuously increased. Enzymes are used as free radical scavengers in sunscreen cream, toothpaste, mouthwashes, hair waving and dyeing (Li et al. 2012). The superoxide dismutase (SOD, EC 1.15.1.1) is used to arrest free radicals and to control damage to skin caused by air and water pollutions, microbes and other harmful factors. SOD and peroxidases are used in combination in sunscreen cream as free radical scavengers to reduce erythema (Babizhayev 2006). Proteases are used in skin creams to clean and smoothen the skin through peeling off dead or damaged skin (Cho et al. 2007).

Other widely used enzymes in toothpaste and mouthwash are endoglycosidase and papain, which are used to whiten teeth, to remove plaque and to remove odor-causing deposits on teeth and gum tissue (Buckingham 1985). Laccase, oxidases, peroxidases, and polyphenol oxidases are used in hair dyeing (Lang and Cotteret 2004), lipase, catalase, papain, bromelain and subtilisin in skin care (Diehl 2008); and protein disulfide isomerase, glutathione sulfhydryl oxidase and transglutaminase in hair waving (Li et al. 2012). Additionally, enzymes are also used in contact lens cleaners to remove protein films (Alfa and Jackson 2001).

### Enzymes in detergents

Detergents represent the largest industrial application of enzymes amounting to 25–30 % of the total sales of enzymes and expected to grow faster at a CAGR of about 11.5 % from 2015 to 2020 (2014c: Global Market for Enzymes in Industrial Applications). Enzymes have contributed significantly to the growth and development of industrial detergents, which is a prime application area for enzymes today. Detergents are used in miscellaneous applications as dishwashing, laundering, domestic, industrial and institutional cleaning (Schafer et al. 2002). The enzymes in detergent products are used to remove protein, starch, oil and fats based stains and to increase the effectiveness of detergents (Kirk et al. 2002; Hasan et al. 2010). The enzymes in laundry detergents are weight efficient, cleave off damaged cotton fibers, improve whiteness, color and fabric care. Enzymes mainly used in detergent products are of hydrolase group and currently, most commonly used enzymes are amylase and protease. Sometimes a combination of enzymes, including proteases, amylases, pectinases, cellulases and lipases used to increase efficiency on stain cleaning and fabric care (Li et al. 2012).

Amylases and lipases are effective on removing starchy food deposits and stains resulting from fatty products, respectively (Masse et al. 2001). Cutinase (EC 3.1.1.74), a hydrolytic enzyme, is used as a lipolytic enzyme in dishwashing and laundry detergents (Filipsen et al. 1998; Pio and Macedo 2009). Protease digests on organic stains, such as grass, blood, egg and human sweat, whereas cellulases are used to brighten colors, soften fabrics and to eliminate small fibers from the fabric without damaging the major fibers of the fabric (Hasan et al. 2010; Kuhad et al. 2011). Protease and amylase are used particularly in dishwasher detergents to remove protein and carbohydrate containing food particles (Keshwani et al. 2015). The application of enzymes in detergent products is advantageous as these products contain less bleaching agents, phosphates, and consequently have beneficial effects on public and environmental health (Olsen and Falholt 1998; Novozyme 2013).

### Organic synthesis industry

Enzyme based processes for production of fine chemicals are rapidly gaining practical significance owing to more economical high purity products in an eco-environmentally acceptable manner (Nagasawa and Yamada 1995). Enzymes are preferred in industrial chemical synthesis over conventional methods for their high selectivity, i.e., chiral, positional and functional group specific (Schmid et al. 2001). Such high selectivity is extremely advantageous in chemical synthesis as it may offer several benefits such as minimal or no by-product formation, easier separation, and less environmental problems. Besides, high catalytic efficiency and mild operational conditions are advantages of enzyme mediated commercial applications. Catalytic potential of microorganisms have been employed for hundreds of years in the production of alcohol, and cheese for industrial synthetic chemistry (Johannes et al. 2006). Among the enzymes in organic synthesis, lipases are the most frequently used, particularly, in the formation of a wide range of optically active alcohols, acids, esters, and lactones (Jaegera and Reetz 1998; Hasan et al. 2006). Lipases are used for the production of (S, R)-2, 3-*p*-ethoxyphenylglycyclic acid, an intermediate for diltiazem (Gentile et al. 1992). Oxidoreductases, such as polyphenol oxidase is involved in the synthesis of 3,4-dihydroxylphenyl alanine (DOPA), a chemical used in the treatment of Parkinson’s disease (Faber 1997). Oligosaccharides and polysaccharides, play vital roles in cellular recognition and communication processes, are synthesized industrially using high regio- and stereoselectivity of glycosyltransferases (Ginsburg and Robbins 1984). Lyases are involved in organic synthesis of cyanohydrins from ketones, acrylamide from acrylonitrile, malic acid from fumaric acid (Faber 1997; Zaks 2001). The nitrile hydratase mediated process for the production of acrylamide is carried out by the Nitto Chemical Company of Japan at a scale of more than 40,000 tons per year (Zaks 2001). A multi-million ton of high fructose corn syrup (HFCS), an alternative sweetener to sucrose in the food and beverage industry, is produced every year commercially using glucose isomerase (Gerhartz 1990).

### Waste treatment

The use of enzyme for waste management is extensive and a number of enzymes are involved in the degradation of toxic pollutants. The industrial effluents as well as domestic waste contain many chemical commodities, which are hazardous or toxic to the living being and ecosystem. Microbial enzyme(s), alone or in combinations, are used for the treatment of industrial effluents containing phenols, aromatic amines, nitriles, etc., by degradation or bioconversion of toxic chemical compound(s) to innocuous products (Klibanov et al. 1982; Raj et al. 2006; Rubilar et al. 2008; Pandey et al. 2011). A number of enzymes employed for waste treatment are amidases, amylases, amyloglucosidases, cellulases, glucoamylases, lipases, nitrile hydratases, pectinases and proteases (Margesin et al.^1999^; Riffaldi et al. 2006; Karigar and Rao 2011). The detoxification of toxic organic compounds through oxidative coupling is mediated with oxidoreductases (Karigar and Rao 2011). These enzymes, like laccase, manganese peroxidase, lignin peroxidase and tyrosinase catalyze the removal of chlorinated phenolic compounds from industrial effluents (Gianfreda et al. 1999; Mai et al. 2000; Have and Teunissen 2001; Piontek et al. 2001; Le Roes-Hill and Prins 2016). The microbial enzymes are also utilized to recycle the waste for reuse, e.g., to recover additional oil from oil seeds, to convert starch to sugar, to convert whey to various useful products (Kalia et al. 2001; http://www.unido.org/fileadmin/import/32068_35FoodWastes). Microbial oxygenases, such as monooxygenases and dioxygenases have a broad substrate range, and are active against a wide range of compounds, including the chlorinated aliphatics (Fetzner and Lingens 1994; Arora et al. 2009). These are used in the degradation of halogenated organic compounds containing pollutants, like herbicides, insecticides, fungicides, hydraulic and heat transfer fluids, plasticizers, and intermediates for chemical synthesis (Fetzner and Lingens 1994; Karigar and Rao 2011).

## Indian enzymes market

Around the globe, enzyme market is dominated by the food and beverage products, and drug industry that go directly or indirectly for human consumption. The biggest challenge in front of fast growing economies such as India is to provide food and healthcare to even their larger population. India, an agriculture-based economy, is predicted to grow at 7.9 % by 2018 (http://data.worldbank.org/country/india) and an attractive market that is opening her doors for industrial enzyme based manufacturing sector. Indian biotech sector accounts 2 % of the global biotech market, but it is gaining worldwide visibility due to the investment opportunities as well as its research output (Binod et al. 2013). Recently, Bharat Biotech, a Hyderabad-based pharma company, has developed world first Zika virus vaccine, which is ready for pre-clinical trials (http://www.huffingtonpost.in/2016/02/07/zika-virus_0_n_9179776.html), demonstrating the “Make in India” efforts (http://www.makeinindia.com/sector/biotechnology). Pharmaceutical enzymes cover almost 50 % of total enzyme demand in India, followed by detergent enzymes (20 %) and textile enzymes (20 %) (Binod et al. 2013).

**Fig. 2:**
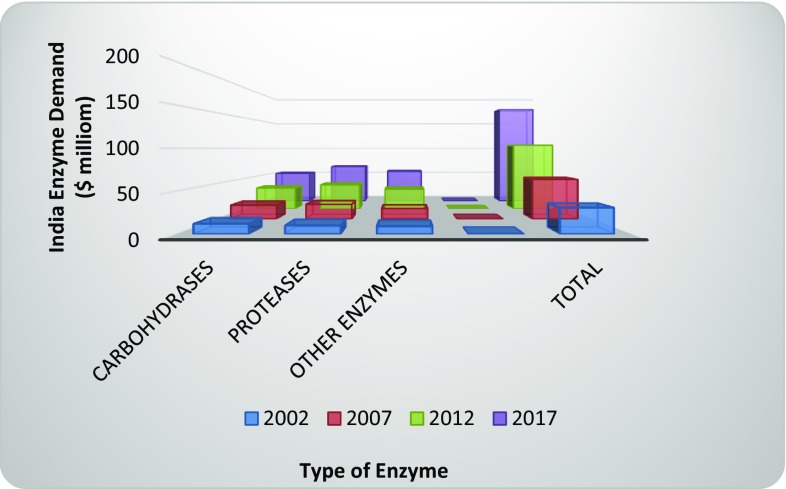
Industrial enzyme demand in India

In 2012, industrial enzymes market globally including the market in India was at marginal position with a net value of around $105 million, but expected to grow significantly with an average of ≥10 % per year through 2017 to reach nearly $173 (http://www.sebi.gov.in/cms/sebi_data/attachdocs/1453372309087.pdf). Greater than 60 % enzyme market in India was contributed by the multinationals companies, whereas the rest was met by the domestic manufacturers. The domestic consumption of enzymes for 2011-12 stood at about $110 million, while the exports raked $32 million in revenues during this period (Biospectrum 2012). In 2012-13, Advanced Enzymes technologies Ltd., the largest manufacturer and exporter of enzyme products in India, had nearly 30 % share in the enzymes industry and second in line after 44 % market share of Denmark based Novozymes. Other prominent manufacturers were Rossari Biotech, Maps Enzymes, Lumis Biotech and Zytex (CRISIL Research 2013) (Table 5). The demand of enzymes by their type is illustrated in Fig. 2 (CRISIL Research 2013), (http://www.sebi.gov.in/cms/sebi_data/attachdocs/1453372309087.pdf).

**Table 5:** Industrial enzyme, manufacturers and market share in India (2012–2013)

Industrial enzymes	Manufacturer	Established	Indian market share (%)	Applications
Protease, xylanase, α-amylases, glucoamylase, etc.	Novozymes India	1983 (Indian manufacturing unit)	44	Household care, textiles, food and beverages, oil and fats, baking, beverage alcohol etc.
Amylase, protease, phytase, xylanase, β-mannanase, α-galactosidase, etc.	Advanced Enzymes technologies Ltd.	1989	30	Food and beverages, pharma, animal feed, textiles, detergent, biofuel, etc.
Amylases, Proteases, Cellulases, Xylanase, Glucoamylase, Pectinase, Catalase, Lipase and Phytase, etc.	MAPS Enzymes Ltd.	1975	Rest of the Market share along with other manufacturers	Textile, leather, feed, etc.
Protease, amylase, cellulase, mannanase, catalase, laccase, pectinases, etc.	Rossari biotech	1997		Textile, pharma, Food and Beverages, feed, detergent, chemical, etc.
Alkaline Pectinase, Amylase, cellulase, Laccase, Catalase, lipase, protease, xylanase, β-glucanase, etc.	Lumisbiotech			Textiles, Food and Beverages, feed, etc.
Protease, pectinase, amylase, xylanase amyloglucosidase, catalases, etc.	Anthem Cellutions (India) Ltd	2007		paper, grain processing, beverages, textiles, baking, animal feed, pharmaceuticals, etc.
Lipase, penicillin amidase, pectinase, Papain,lysozyme, etc.	Aumgene Biosciences	2004		Textile, pharma, Food and Beverages, feed, detergent, etc.
Nattokinase, phytase, lipase amylase, protease, cellulase, etc.	Zytex India Pvt. Ltd.	1947 (1st manufacturing unit in 1996)		Textile, food, nutraceuticals, animal feed, etc.

## Conclusion

The prospects of industrial uses of microbial enzymes have increased greatly in 21st century and continuously increasing as enzymes have significant potential for many industries to meet demand of rapidly growing population and cope exhaustion of natural resources. It had been showed here that the enzymes have enormous potential in various industrial sectors that may be pharmaceuticals, food, feed, beverages, detergents, leather processing and paper & pulp. Alternatively, these biomolecules may be used consistently to meet continuously rising demand of food supply. Enzymes of microbial origin have significant potential in waste management, and consequently in the development of green environment. The enzymes are effectively utilized in many industries for higher quality productions at accelerated rate of reaction with innocuous pollution and cost effectiveness.
